# Genome-wide patterns of selection–drift variation strongly associate with organismal traits across the green plant lineage

**DOI:** 10.1101/gr.279002.124

**Published:** 2024-08

**Authors:** Kavitha Uthanumallian, Andrea Del Cortona, Susana M. Coelho, Olivier De Clerck, Sebastian Duchene, Heroen Verbruggen

**Affiliations:** 1 Melbourne Integrative Genomics, School of BioSciences, University of Melbourne, Parkville VIC 3010, Australia;; 2 Department of Biology, Phycology Research Group, Ghent University, 9000 Ghent, Belgium;; 3 Department of Algal Development and Evolution, Max Planck Institute for Developmental Biology, 72076 Tübingen, Germany;; 4 Department of Microbiology and Immunology, Peter Doherty Institute for Infection and Immunity, University of Melbourne, Parkville VIC 3010, Australia;; 5 Department of Computational Biology, Institut Pasteur, 75015 Paris, France;; 6 CIBIO, Centro de Investigação em Biodiversidade e Recursos Genéticos, InBIO Laboratório Associado, Campus de Vairão, Universidade do Porto, 4485-661 Vairão, Portugal

## Abstract

There are many gaps in our knowledge of how life cycle variation and organismal body architecture associate with molecular evolution. Using the diverse range of green algal body architectures and life cycle types as a test case, we hypothesize that increases in cytomorphological complexity are likely to be associated with a decrease in the effective population size, because larger-bodied organisms typically have smaller populations, resulting in increased drift. For life cycles, we expect haploid-dominant lineages to evolve under stronger selection intensity relative to diploid-dominant life cycles owing to masking of deleterious alleles in heterozygotes. We use a genome-scale data set spanning the phylogenetic diversity of green algae and phylogenetic comparative approaches to measure the relative selection intensity across different trait categories. We show stronger signatures of drift in lineages with more complex body architectures compared with unicellular lineages, which we consider to be a consequence of smaller effective population sizes of the more complex algae. Significantly higher rates of synonymous as well as nonsynonymous substitutions relative to other algal body architectures highlight that siphonous and siphonocladous body architectures, characteristic of many green seaweeds, form an interesting test case to study the potential impacts of genome redundancy on molecular evolution. Contrary to expectations, we show that levels of selection efficacy do not show a strong association with life cycle types in green algae. Taken together, our results underline the prominent impact of body architecture on the molecular evolution of green algal genomes.

A growing body of evidence supports the idea that organismal traits, particularly structural complexity and life cycle traits, can influence molecular evolution (Bromham et al. 1996, 2015; Otto and Gerstein 2008; Thomas et al. 2010; Figuet et al. 2016). Structural complexity has evolved from small, structurally less complex (unicellular) ancestors to large, more complex (multicellular) forms on many occasions (Cock et al. 2014; Sebé-Pedrós et al. 2017; De Clerck et al. 2018; Umen and Herron 2021). An increase in structural complexity is associated with increases in body mass and size, which usually coincides with a substantial reduction in effective population size (*N*_e_) in structurally complex forms compared with simple unicellular forms (Fig. 1; Romiguier et al. 2014; Ellegren and Galtier 2016; Herrera-Álvarez et al. 2021). *N*_e_ is a crucial factor for determining the evolutionary fate of new mutations, altering the efficacy of selection relative to drift (Ohta 1972, 1992; Charlesworth 2009; Chen et al. 2017), an idea supported by studies showing that populations with larger *N*_e_ experience stronger selection efficacy (Lynch and Conery 2003; Gossmann et al. 2012).

**Figure 1.**
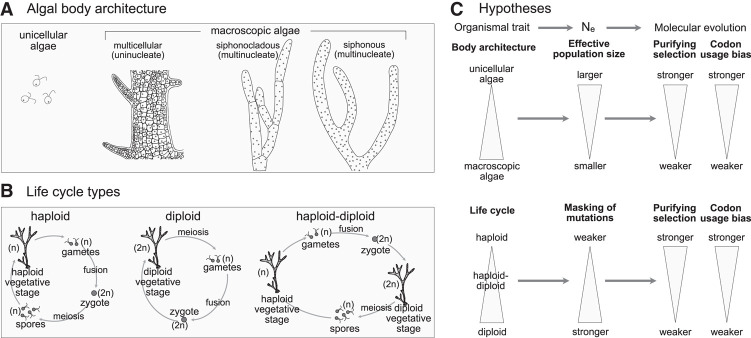
Variation in algal body architecture (*A*) and life cycles (*B*), as well as our working hypotheses of how these traits impact on molecular evolution (*C*). Reduced effective population sizes associated with increasing complexity of body architecture are likely to decrease the levels of purifying selection. For life cycles, haploid-dominant lineages are expected to have stronger selection than diploid-dominant lineages as a consequence of the masking of silenced mutations.

Similarly, an organism's life cycle type may influence its molecular evolution. Three basic life cycle types have evolved repeatedly among eukaryotes (Fig. 1B). In the haploid cycle, the principal life stage is haploid, and diploidy only exists in the zygote. In the diploid cycle, the principal life stage is diploid, and haploidy only exists in the gametes. In the haploid–diploid cycle, mitosis occurs both in diploid and haploid phases, resulting in haploid and diploid multicellular life stages. Theoretical models suggest that haploid organisms should experience stronger selection efficacy as they have only one copy of the genetic material, leading to effective elimination of deleterious mutations, whereas in diploid organisms, recessive deleterious mutations can be masked by the other allele and may therefore accumulate in the genome (Mable and Otto 1998; Otto and Gerstein 2008). Although several features associated with life cycles, like the proportion of time spent in each stage of the life cycle and mode of reproduction, can impact the efficacy of selection, this information is not known for most species (Otto and Marks 1996). At present, there is little empirical evidence for a correlation between life cycle types and molecular evolution (Heesch et al. 2021; Krasovec et al. 2023). It is important to note that life cycles and structural complexity are often correlated, with diploid life cycles (or haploid–diploid cycles with a dominant diploid generation) occurring more frequently in organisms with higher levels of structural complexity (Coelho et al. 2007).

In this study, we investigate the association of structural complexity and life cycle types on molecular evolution in the green lineage (Viridiplantae), with an emphasis on the green algae. The Viridiplantae span a long evolutionary history of nearly 1.5 billion years (Leliaert et al. 2012; Hou et al. 2022) and contain more than 22,000 species of green algae (Guiry 2012), including the algal ancestors from which a large diversity of land plants has evolved (Wang et al. 2020). Green algae feature a broad range of structural complexity, from unicellular and colonial algae to various more complex body architectures in the green seaweeds and land plants. We will use the term “body architecture” to indicate the cytomorphological diversity exhibited by algae. The green seaweeds (class Ulvophyceae) present a variety of body architectures (Fig. 1A), including unicellular and multicellular forms. Such multicellular forms are often called thalli and evolved independently from those seen in land plants. Also, some species have siphonocladous structure, in which the algal body is made up of very large multinucleated cells with regularly spaced individual nuclei, lacking cytoplasmic streaming (Mine et al. 2008). Lastly, the siphonous algae consist of a single, giant, branched tubular cell with thousands of nuclei, in many cases showing subcellular morphological and functional differentiation (Cocquyt et al. 2010; Del Cortona and Leliaert 2018; Umen and Herron 2021). This diversity of body architectures along with the observed variation in life cycle types highlight the Viridiplantae as an excellent model to study the association of these traits with molecular evolution.

Our work is centered on evaluating the predictions of the near-neutral theory (Ohta 1996) and the masking effect on lineages with different body architecture and life cycle types. Based on these theoretical expectations, we hypothesize associations between organismal traits and patterns of molecular evolution (Fig. 1C). For algal body architecture, we anticipate that the larger *N*_e_ of unicellular algae compared with macroscopic algae categories will lead to stronger levels of selection. We have no a priori expectation for differences between the different architectures among macroscopic algae, as each of these categories includes seaweeds of similar body size and hence, arguably, similar *N*_e_. The genetic redundancy following from multinucleated cells and endopolyploidy in siphonous and siphonocladous algae (Kapraun 1994) leads us to speculate that their patterns of molecular evolution may differ from those of unicellular and traditional multicellular algae.

As for life cycle types, we anticipate haploid lineages to experience more purifying selection owing to the haploid life stage being prone to elimination of deleterious mutations (Orr and Otto 1994; Gerstein et al. 2011). Diploid lineages are expected to sit on the opposite end of the spectrum, experiencing more drift, and lineages with haploid–diploid cycles can be expected to fit in between these extremes (Stoeckel et al. 2021).

## Results

### Experimental design

**Figure 2.**
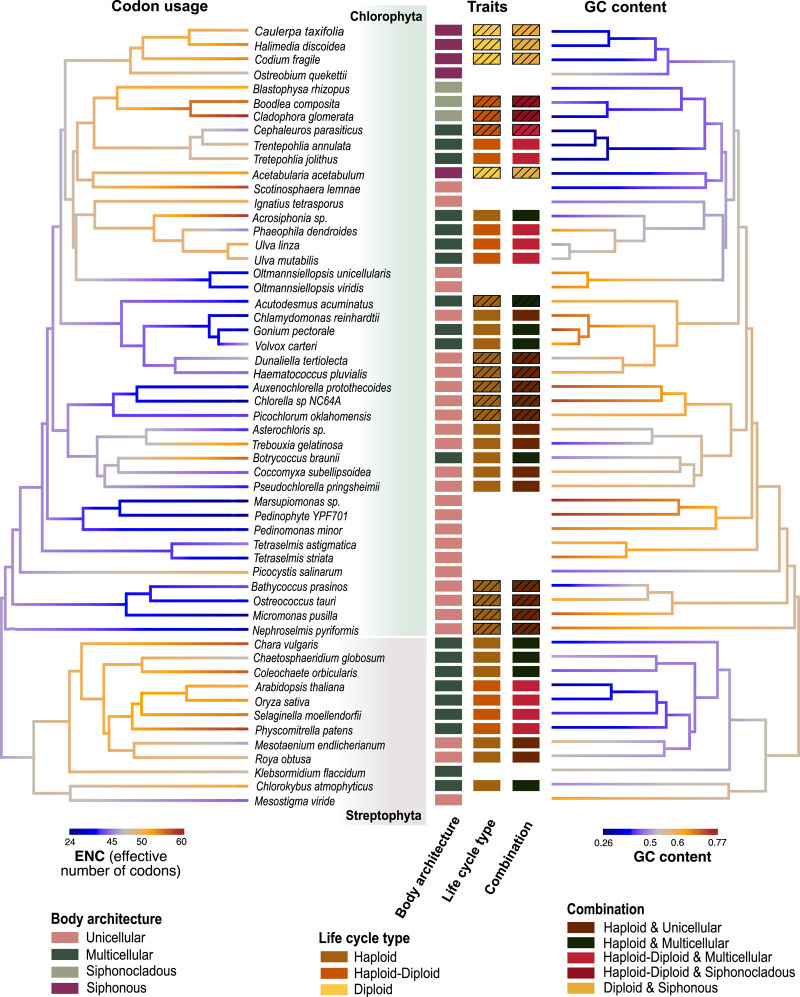
Phylogeny illustrating evolutionary relationships, trait values, codon usage, and GC content across green algae. Green algal phylogeny is mapped with effective codon bias (ENC) and GC content. The *center* panel boxes following the names of the lineages are colored based on trait values and indicate the different categories these lineages represent in the three models: body architecture, life cycle types, and the combined (body architecture + life cycle type) models. The conservative life cycle information is indicated by plain colored boxes; extrapolated information from the more liberal data set, using boxes outlined by black borders. The phylogeny is drawn with a blue–orange gradient indicating codon bias (ENC; *left*) and GC content (*right*), showing that these traits are interrelated, as most unicellular lineages exhibit higher codon bias (lower ENC; blue) and higher GC content (orange) relative to other lineages.

By implementing phylogenetic comparative approaches, we tested the hypotheses about molecular evolution using a densely sampled, genome-wide data set and phylogenetic tree of green algae (Fig. 2; Supplemental Table S1). We evaluate selection intensity variation (estimated as rates of synonymous and nonsynonymous substitution and as codon bias) using three evolutionary models (M1, M2, and M3). The models are based on body architectures (M1) with the categories unicellular (U), multicellular (M), siphonous (Sp), and siphonocladous (Sc); life cycles (M2) with the categories haploid (H) and haploid–diploid (HD); and combined traits (M3) with the categories unicellular haploid (UH), multicellular haploid (MH), and multicellular haploid–diploid (MHD).

The strength of selection was measured by omega, the ratio of nonsynonymous (*d*_N_) to synonymous substitutions (*d*_S_), obtained using a variable-ratio branch model. Besides omega values, we also dissect the signal from *d*_S_ and *d*_N_ substitutions separately to get more detailed insights into neutral and selection-driven substitutions.

### Single-copy housekeeping genes evolve under strong purifying selection

**Figure 3.**
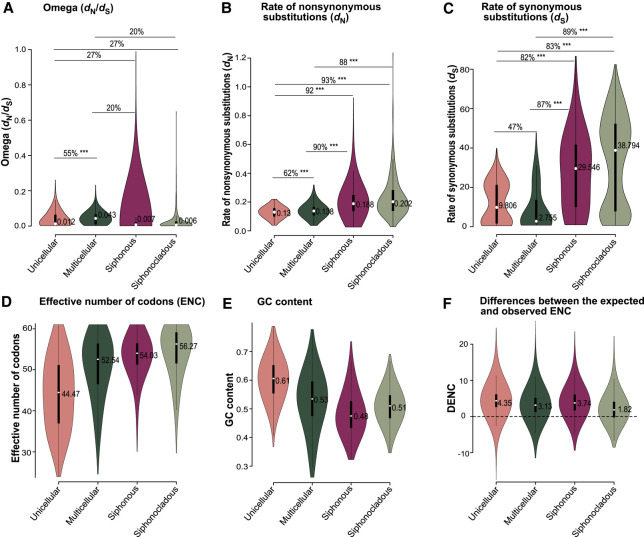
Association of body architecture with molecular evolution. The violin plots show the distribution of omega (*A*), rates of nonsynonymous substitutions (*B*), rates of synonymous substitutions (*C*), effective number of codons (*D*), GC content (*E*), and the difference between the expected and observed ENC (*F*) for four body architecture categories. Median values are listed in the violin plot. The horizontal lines refer to comparisons across body architecture types, with indications of the percentages of genes following expected patterns in these comparisons, and the significance of the differences as given by *P*-values of the gene-by-gene Wilcoxon test. (*) *P* ≤ 0.05, (**) *P* ≤ 0.001, (***) *P* ≤ 0.0001. The lower values of *d*_N_ and ENC suggest stronger selection intensity in unicellular algae relative to other algae with complex body structures.

**Figure 4.**
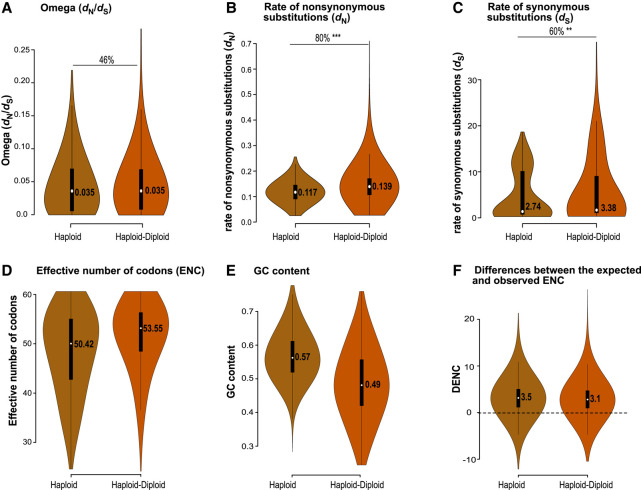
Association of life cycle types with molecular evolution. The violin plots show the distribution of omega (*A*), rates of nonsynonymous substitutions (*B*), rates of synonymous substitutions (*C*), effective number of codons (*D*), GC content (*E*), and the difference between the expected and observed ENC (*F*) for lineages with haploid and haploid–diploid life cycles, based on the conserved data set described in the text. Median values are listed in the violin plot. The horizontal lines refer to comparisons across body architecture types, with indications of the percentages of genes following expected patterns in these comparisons, and the significance of the differences as given by *P*-values of the gene-by-gene Wilcoxon test. (*) *P* ≤ 0.05, (**) *P* ≤ 0.001, (***) *P* ≤ 0.0001. The lower values of *d*_N_ , *d*_S_, and ENC suggest stronger selection intensity in haploid than haploid–diploid algal lineages.

**Figure 5.**
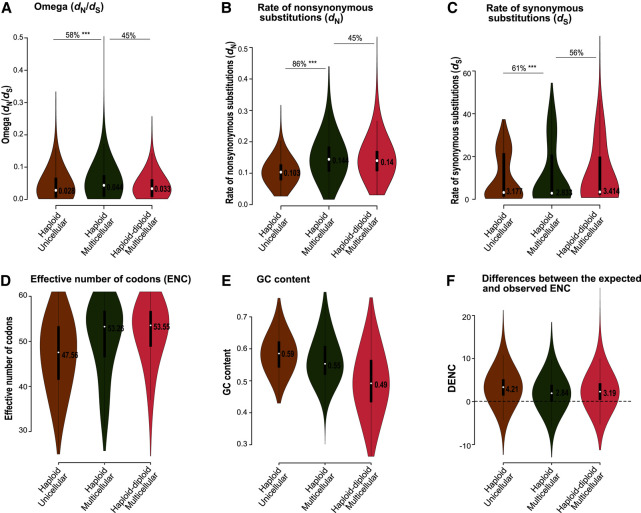
Combined effect of body architecture and life cycle types on molecular evolution. The violin plots show the distribution of omega (*A*), rates of nonsynonymous substitutions (*B*), rates of synonymous substitutions (*C*), effective number of codons (*D*), GC content (*E*), and the difference between the expected and observed ENC (*F*) for three combinations of body architecture and life cycle type. The horizontal lines refer to comparisons across body architecture types, with indications of the percentages of genes following expected patterns in these comparisons, and the significance of the differences as given by *P*-values of the gene-by-gene Wilcoxon test. (*) *P* ≤ 0.05, (**) *P* ≤ 0.001, (***) *P* ≤ 0.0001. The lower values of *d*_N_ and ENC suggest stronger selection intensity in unicellular haploid relative to other categories, and highly similar values of *d*_N_ and ENC between multicellular haploid and haploid–diploid lineages suggest similar levels of selection between these groups.

To enable comparison of genes across distantly related lineages, our analysis uses single-copy housekeeping genes. We expect these to be evolving under purifying selection, and our design investigates differences in the level of relaxation of this purifying selection. The omega values inferred from branch models were well below one across genes (Figs. 3A, 4A, 5A; Supplemental Figs. S5A, S6A), confirming the overall purifying selection acting on these conserved genes. Low omega values (less than one) indicate that substitutions are less frequent at selected sites (*d*_N_) relative to neutral sites (*d*_S_), indicating stronger purifying selection.

### Unicellular algae accumulate fewer deleterious mutations

The body architecture models (M1) showed that unicellular lineages had lower omega values than multicellular lineages, and the difference between them was significant, but only 55% of studied genes showed this trend (Fig. 3A; Supplemental Table S2). Siphonous and siphonocladous lineages had low median omega values, which we will address in more detail below.

Separate plots of rates of nonsynonymous (Fig. 3B) and synonymous substitutions (Fig. 3C) show that macroscopic algal lineages (multicellular, siphonous, and siphonocladous) feature higher levels of nonsynonymous substitutions than unicellular lineages (Fig. 3B), suggesting increased drift in these more complex organisms. The rates of synonymous substitutions of unicellular algae had a broad distribution, with a median higher than multicellular algae (Fig. 3C) but with very similar proportions of genes showing lower versus higher *d*_S_ (47% vs. 53%) between these two groups.

### Codon bias and GC content confirm strong selection in unicellular algae

We investigated levels of codon usage bias across species with different trait values, as it reflects the role of mild selection on synonymous sites (Akashi 1995, 1997; Subramanian 2008) and has not been used much to evaluate near-neutral predictions. The results of nucleotide compositional analysis showed that unicellular algae had stronger GC bias (median, 0.61) than macroscopic algae (median, ∼0.5) (Fig. 3E), corresponding with a reduced selection intensity in the macroscopic algae as suggested by their lower codon bias (median ENC, >50) relative to unicellular algae (median ENC, 44.47) (Fig. 3D). GC content was thus clearly associated with stronger codon usage bias (Figs. 2, 3D, 3E; Supplemental Fig. S1), and the strong positive correlation between the probability of overall GC content with the use of GC in the degenerative positions of codons for amino acids with four and six degenerative codons suggests that compositional bias drives codon usage (Supplemental Figs. S2, S3).

The difference between EENC and OENC indicates that factors other than compositional bias may also contribute to codon usage. However, the small DENC across all species (Figs. 3F, 4F, 5F; Supplemental Figs. S4, S5F, S6F; Supplemental Table S3) along with the strong association between overall GC content and codon usage for four- and sixfold degenerative codons suggest that GC compositional constraint is the dominant factor in shaping macroevolutionary trends in codon usage across the green algal phylogeny.

### Siphonous and siphonocladous algae are remarkable outliers

Contrary to our theoretical expectation, the macroscopic siphonous and siphonocladous algae featured the lowest omega values (Fig. 3A), at first sight suggesting stronger selection efficacy in these groups. However, the plots of *d*_N_ and *d*_S_ separately suggest a different story, as the siphonous and siphonocladous algae are clear outliers with substantially elevated rates of substitutions (Fig. 3B,C). The low omega values are thus likely to be a consequence of high rates of synonymous substitutions overshadowing the elevated nonsynonymous substitutions. When considering all evidence together, the higher rates of synonymous and nonsynonymous substitutions, weaker codon bias, and AT-biased genes (Fig. 3B,D,E) strongly suggest the siphonous and siphonocladous algae have elevated drift compared to unicellular algae.

### Haploid–diploid algae accumulate more mutations

Because we did not have published information for life cycle types across all species, we designed two data sets (Fig. 2; Supplemental Table S1). The conservative data set contained only those taxa for which there is published information on their life cycles. For the liberal data set, we inferred life cycle types for some species based on published information of related species or published interpretations by experts. Our evolutionary models based on life cycle types (M2) showed that haploid and haploid–diploid lineages had similar omega values (Fig. 4A; Supplemental Table S4), suggesting roughly equivalent levels of selection. Higher rates of *d*_N_ and *d*_S_ for haploid–diploid lineages relative to haploid lineages (Fig. 4B,C), however, underline the possibility of elevated drift in haploid–diploid lineages, in line with theoretical expectations. Lower ENC and higher GC content relative to haploid–diploid lineages also suggest that haploid lineages may experience stronger selection efficacy (Fig. 4D,E). The liberal data set confirmed the results of the conservative data set and, in addition, shows increased drift in diploid lineages (Supplemental Fig. S5; Supplemental Table S5).

The results from combined trait model (M3) show that between unicellular and multicellular algae with the same life cycle (haploid), the unicellular algae have higher levels of purifying selection, based on their lower values of *d*_N_, *d*_S_, ENC, and GC content (Fig. 5B–E; Supplemental Table S6). When comparing algae that have the same body architecture (multicellular) but differ in life cycles (haploid vs. haploid–diploid), our results show similar levels of purifying selection, with comparable omega, *d*_N_, and *d*_S_ distributions and nearly identical ENC (Fig. 5D), implying that life cycle type per se does not correlate strongly with molecular evolution in our data set. The results based on the more liberal data set are in line with those of the conservative data set and, in addition, suggest that multicellular haploid lineages had lower rates of substitutions than multicellular haploid–diploid lineages (Supplemental Fig. S6; Supplemental Table S7).

## Discussion

### Body architecture strongly correlates with molecular evolution

The influence of effective population size on the efficacy of purifying selection, imposing constraints on amino acid-altering nonsynonymous substitutions, is supported by both theoretical and empirical work (Ohta 1992; Moran 1996; Woolfit and Bromham 2005; Lanfear et al. 2014; Lynch et al. 2016). Our results are in line with this reasoning, as unicellular algae can have very large populations in the phytoplankton (Blanc-Mathieu et al. 2017; Krasovec et al. 2017; Rengefors et al. 2017), and the stronger drift in macroscopic lineages is probably owing to a decrease in the efficacy of purifying selection in the comparatively small populations of macroalgae (Van Der Strate et al. 2002; Coyer et al. 2008; Jueterbock et al. 2018).

Theory predicts that synonymous substitutions are effectively neutral and that their rate of fixation is less likely to be driven by selection. Thus, the rate of synonymous substitutions is less likely to correlate with *N*_e_ and instead reflects the underlying mutation rate (Ohta and Kimura 1971; Ohta 1972). Based on this, we speculate that accumulation of effectively neutral mutations indicated by our results in unicellular algae relative to macroscopic algae might be owing to generation-time effects on synonymous sites relative to nonsynonymous sites (Ohta 1992, 1995). The generation-time effect is mainly because of the impacts of frequency of DNA replication on accumulation of copy errors (Bromham et al. 1996; Bromham 2002), and unicellular algae likely experience higher rates of DNA replication per unit time owing to shorter generation times.

A trend toward lower GC content in more complex algae is observed across independent gains of complexity in the streptophytes and chlorophytes (Fig. 2), suggesting it is a fairly general trend (Buschmann 2020). Although increase in GC content can also be attributed to processes such as GC-biased gene conversion, there is strong evidence from studies on the genomes of endosymbionts, prokaryotes, and eukaryotes that mutation is biased toward AT enrichment with increased drift (Lynch and Conery 2003; Balbi et al. 2009; Hershberg and Petrov 2010; Lynch 2010b; McCutcheon and Moran 2012). Following that reasoning, higher AT content in macroscopic algae relative to unicellular algae may be owing to increased drift in the arguably smaller macroalgal populations.

Our findings of a strong association between GC content and codon usage bias are in accordance with previous studies (Chen et al. 2004; LaBella et al. 2019). In our case, GC compositional bias appears to be the main factor influencing codon usage bias. We emphasize that these insights are for the macroevolutionary inferences we make here on a set of conserved green algal genes, and further insights into translational selection on codon usage can be gained by carrying out gene-level comparisons within individual genomes (Naya et al. 2001; Michely et al. 2013; Yao et al. 2023). As codon usage is often influenced by tRNA and gene expression levels (Plotkin and Kudla 2011; Wint et al. 2022), further studies on correlating the green algal tRNA populations and codon usage may provide insights into the evolution of selection pressure related to translational efficiency in green algae.

Our results highlight the elevated drift in siphonous and siphonocladous algae, and we argue that it may be a consequence of their peculiar nature, with large multinucleate cells and several copies of the genetic material (endopolyploidy, i.e., somatic variation in ploidy levels) (Kapraun 1994, 2005, 2006; Del Cortona and Leliaert 2018). These features could result in redundant copies of genes, possibly allowing the accumulation of mutations silenced by masking (Comai 2005; Otto 2007). At the molecular level, increased rates of mutations can be attributed to error-prone polymerases and repair mechanisms decreasing the fidelity of DNA replication (Denver et al. 2006; Lynch 2010a; Belfield et al. 2018). Knowledge on DNA repair mechanisms in siphonous or siphonocladous algae is very limited (Repetti et al. 2020), but it could be argued that genetic redundancy in multinucleate cells may go hand in hand with more liberal DNA repair, enhancing the accumulation of mutations relative to algae with other body architectures, which would explain the increased drift in multinucleate organisms observed in our results.

Signatures of stronger selection efficacy in unicellular algae relative to more complex macroalgal forms establish effective population size as an important factor in algal molecular evolution. This is in agreement with the near-neutral theory, which posits *N*_e_ as a prime determinant of molecular evolution. Yet, it is clear from our results that other factors that covary with body architecture can also affect molecular evolution, particularly the striking difference in the molecular evolutionary trends of siphonous and siphonocladous algae. Overall, our observations on molecular evolution, along with the interesting cellular and nuclear features of the siphonous and siphonocladous algae, highlight their potential role as a model for the study of selection–drift balance and genome maintenance.

### Life cycle types do not correlate strongly with molecular evolution

In line with our expectations, we observed stronger selection in haploid than in haploid–diploid lineages. However, in these interpretations we must take into account the correlation of life cycle types and morphological types. The haploid lineages include unicellular (N = 7) and multicellular (N = 8) species, whereas the haploid–diploid lineages are all multicellular algae (Fig. 4; Supplemental Table S3). So, the stronger selection of haploid lineages may, in part, result from the larger effective population size in unicellular algae. An important caveat with the observation of life cycle–based models for the liberal data set is that all diploid lineages in the data set are siphonous algae (Supplemental Fig. S5; Supplemental Table S5); hence, any effect of masking owing to the diploid life cycle may be obscured by the effect of lower *N*_e_ of this macroalgal group.

To separate the effects of life cycles and body architecture, we designed models that accommodate combinations of traits (M3). The results of these models reinforced the effect of body architecture as an important correlate of molecular evolution. Here it should be considered that the haploid multicellular category includes simple colonial organisms like *Volvox*, *Gonium*, and *Botrycoccus* and that multicellular haploid–diploid lineages are often more complex multicellular organisms in the Trentepohliales, Ulvophyceae, and streptophytes, so the effect of the larger population sizes for colonial forms may contribute to the observed trend.

All evidence taken together, for our data set, life cycle types contribute less to variation in patterns of molecular evolution than body architecture. Although our data allowed making clear inferences for haploid versus haploid–diploid life cycles, we could not test for the stronger levels of masking expected in fully diploid lineages. This was because of the diploid species in our data set all being siphonous, limiting our ability to separate effects of body architecture from those of masking. We concur with Otto and Marks (1996) that future studies should focus on lineages with additional diversity in life cycle, including a strong representation of diploid lineages to provide a more comprehensive test of the masking hypothesis.

## Methods

### Model system and data

The molecular data set used for this study contains well-conserved 539 single-copy nuclear genes from 55 green algal lineages, as well as a tree inferred from a previous study (Fig. 2; Supplemental Table S1; Del Cortona et al. 2020). These genes were obtained by filtering for high-confidence genes from 620 picoPLAZA single-copy genes (Vandepoele et al. 2013). The steps involved in extracting CDS from genomes and transcriptomes and obtaining the 539 single-copy nuclear genes are described in previous work (Del Cortona 2018, 2020). The putative functions of these 539 single-copy gene groups were predicted using the annotations of *Chlamydomonas reinhardtii* and *Arabidopsis thaliana* sequences in each of these groups (Supplemental Table S8).

We divided the species into four categories representing different algal body architectures (Fig. 1A): 27 unicellular algae, 20 multicellular species that include multicellular seaweeds that are uninucleate but also land plants (N = 4) and colonial algae (N = 2), three siphonocladous species, and five siphonous species (Supplemental Table S1; Supplemental Fig. S7). The body architecture categories that we employed were fairly broad, with multicellular algae, for instance, including both small colony-forming algae all the way to much larger land plants. Our goal was to choose categories so as to retain sufficient taxon sampling within each category, because the number of taxa for which extensive nuclear genomic data sets are available remains rather small.

The conservative data set for life cycles contains 15 haploids, nine haploid–diploids, and one diploid, discarding 30 species for which there was no published information on life cycle types (Supplemental Table S1; Supplemental Fig. S8). We decided to also exclude the sole diploid species to avoid issues of sampling bias and inference precision. We also removed the siphonous and siphonocladous species from this data set, because the interpretation of life cycles, particularly in many siphonous green algae, is problematic (Hoek et al. 1995) and because the strong association of molecular evolutionary patterns with these body architectures (see results) might prevent detecting any effects of life cycles on molecular evolution. Our second data set is a more liberal data set containing 25 haploid, four diploid, and 12 haploid–diploid species (Supplemental Table S1; Supplemental Fig. S10).

### Evolutionary models to compare levels of purifying selection across traits

We used the variable-ratio branch model of CODEML from PAML v 4.9i that uses a codon-based likelihood approach that can model different selection intensities for categories of branches in a phylogeny corresponding to a mapped trait (Yang 1998; Yang and Nielsen 1998; Yang 2007). We ran PAML on the multiple sequence alignments for each gene to obtain estimates of the rates of nonsynonymous (*d*_N_) and synonymous (*d*_S_) substitutions and their ratio (omega, *d*_N_/*d*_S_). Branch categories corresponded to inferred ancestral states of body architecture and life cycle types. The selection intensity is estimated for each of the branch categories corresponding to the organismal trait categories of the models.

Our first model (M1: U, M, Sp, Sc) aims to detect selection intensity variations in algal lineages with different body architecture. The branches of the phylogeny were categorized as one of the four body architectural categories (unicellular [U], multicellular [M], siphonous [Sp], and siphonocladous [Sc]), based on the ancestral character states estimated by Del Cortona et al. (2020). The rate of nonsynonymous substitution (*d*_N_), rate of synonymous substitution (*d*_S_), and omega (*d*_N_/*d*_S_) were inferred for each of the four categories by allowing four different selection patterns corresponding to the trait categories (Supplemental Fig. S7). To evaluate the statistical significance of differences in molecular evolutionary parameters between unicellular algae and the three types of macroscopic algae, a nonparametric paired Wilcoxon signed-rank test was used. The data points for this test are the *d*_N_, *d*_S_, or *d*_N_/*d*_S_ values inferred for the four different body architectures for each of the genes. Each test compares the values for a single substitution feature (e.g., *d*_N_) across the different body architectures for all genes simultaneously. In situations in which molecular data were missing for a gene, the taxa with missing data were pruned from the tree prior to PAML analysis.

The second model (M2: H, HD) investigates selection intensity variation for lineages with different life cycle types (Supplemental Fig. S8). For this analysis, the phylogeny was pruned to the taxa for which life cycle information was available, and haploid (H) and haploid–diploid (HD) branches were determined by ancestral state estimation using stochastic mapping with the phytools R package (Revell 2012; R Core Team 2018).

Because structural complexity and life cycles correlate to some extent, we designed a model (M3: UH, MH, MHD) with categories representing combinations of traits: unicellular haploid (UH), multicellular haploid (MH), and multicellular haploid–diploid (MHD) branches (Supplemental Fig. S9). From the relative selection intensity of unicellular and multicellular haploid, we can isolate the impacts of body architecture as they have similar life cycles. Similarly, comparison of the multicellular haploid and multicellular haploid–diploid lineages allows us to isolate the impacts of life cycle types in multicellular lineages.

Equivalent models for the liberal life cycle types data set are described in the Supplemental Material (Supplemental Figs. S10, S11; Supplemental Text S1).

### Codon usage bias

A growing body of evidence indicates that synonymous substitutions are not fully neutral and are not entirely determined by underlying rates of mutations (Stoletzki and Eyre-Walker 2007; Plotkin and Kudla 2011). We quantified codon usage bias as an estimate of selection efficacy on synonymous sites. We calculated the effective number of codons (ENC) for each species using the ENC method from coRdon (https://github.com/BioinfoHR/coRdon). ENC values range from 20, indicating strong codon usage bias in which only a single codon is used per amino acid, to 61, when all synonymous codons are equally used for each amino acid (Wright 1990). Lower ENC signifies constrained use of codons, that is, stronger codon bias, which reflects an underlying trend of stronger selection at synonymous sites.

ENC values were estimated with the standard genetic code except for taxa that use the dasycladacean nuclear code, namely, the Trentepohliales (*Trentepohlia jolithus*, *Theileria annulata*, *Cephaleuros parasiticus*), the Cladophorales (*Cladophora glomerata*, *Boodlea composita*), *Blastophysa rhizopus,* the Dasycladales (*Acetabularia acetabulum*), and the Scotinospaerales (*Scotinosphaera lemnae*) (Del Cortona et al. 2020).

Because nucleotide composition and codon bias are interrelated, we used a method allowing us to estimate the influence of nucleotide composition on codon usage bias. We calculated the overall GC composition and GC at the third codon position (GC3) for all the sequences. From this, the theoretically expected ENC (EENC) values based on the GC3 composition were calculated (Wright 1990), and the difference (DENC) between the expected ENC and the observed ENC (OENC; estimated from the gene sequence) was calculated (DENC = EENC – OENC). A value of zero for DENC indicates that codon bias is entirely determined by nucleotide composition, whereas a positive DENC implies a role of selection constraints at the translation level beyond the influence of nucleotide composition.

## Data access

The derived data sets generated in this study, including multiple sequence alignments of the single-copy genes and the phylogenetic trees corresponding to different models, are available at Zenodo (https://doi.org/10.5281/zenodo.11435053) and as Supplemental Data.

## Supplementary Material

Supplement 1

Supplement 2

Supplement 3

Supplement 4

Supplement 5

Supplement 6

Supplement 7

Supplement 8

Supplement 9

Supplement 10

Supplement 11

Supplement 12

Supplement 13

Supplement 14

Supplement 15

Supplement 16

Supplement 17

Supplement 18

Supplement 19

Supplement 20

Supplement 21
