# Supplementary material for: Genome-wide patterns of selection–drift variation strongly associate with organismal traits across the green plant lineage
Source: Genome Res. 2024 Aug;34(8):1130–9. doi: 10.1101/gr.279002.124 (PMC11444171; doi:10.1101/gr.279002.124)
Supplement: Supplement 1 [file Supplemental_figure_S1.pdf]

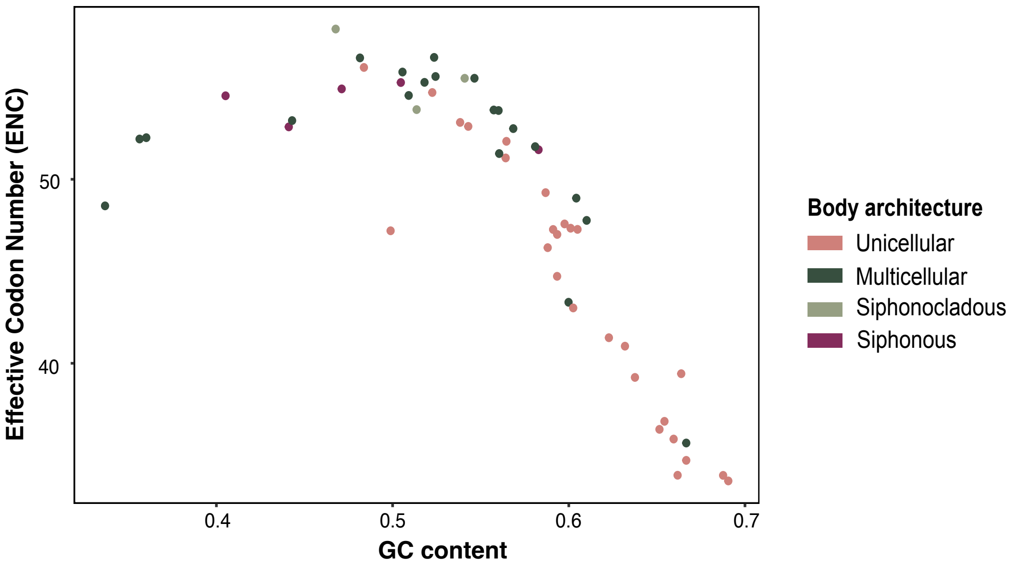

Supplemental Figure S1: Association of ENC and GC across the green algal phylogeny. ENC variation is highly correlated with GC3 across the green algal phylogeny. In this plot organisms with higher ENC value occupied middle area of the curve and organisms that fall in the GC/AT-rich area had lower ENC, highlighting compositional constraint as a probable governing factor.
