# Supplementary material for: Genome-wide patterns of selection–drift variation strongly associate with organismal traits across the green plant lineage
Source: Genome Res. 2024 Aug;34(8):1130–9. doi: 10.1101/gr.279002.124 (PMC11444171; doi:10.1101/gr.279002.124)
Supplement: Supplement 3 [file Supplemental_figure_S3.pdf]

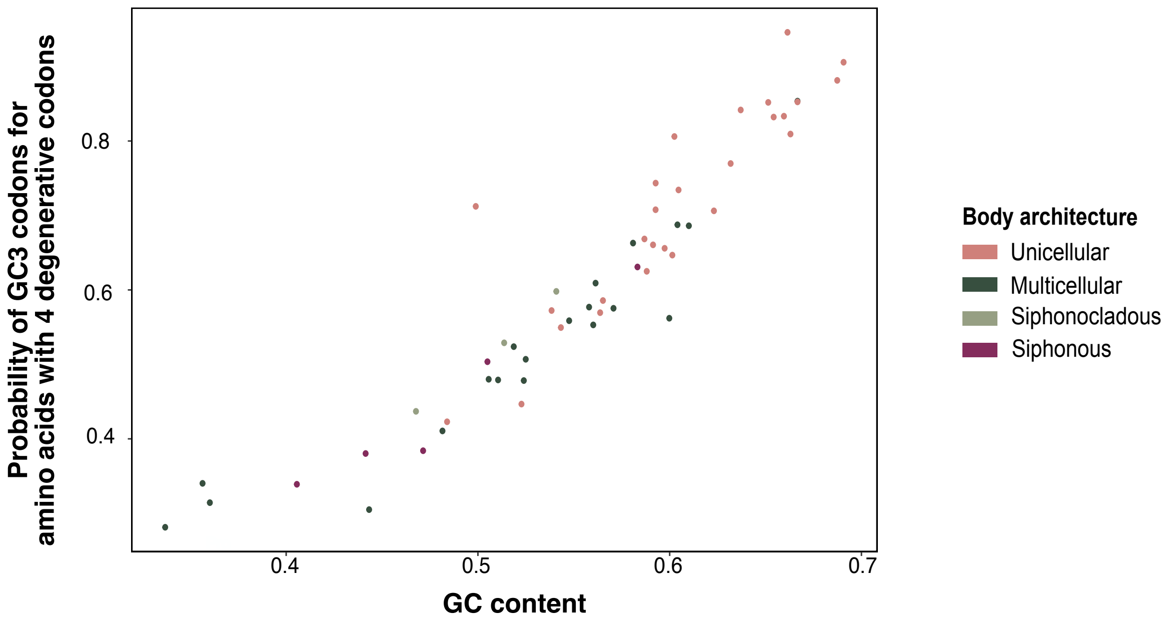

Supplemental Figure S3: Plot showing the correlation between the probability of GC3 codons for amino acids with 4 degenerative codons and GC content. The linear correlation between the probability of GC3 codons for aminoacids with 4 degenerative codons and GC content suggests that GC composition is major factor driving usage of these codons.
