# Supplementary material for: Genome-wide patterns of selection–drift variation strongly associate with organismal traits across the green plant lineage
Source: Genome Res. 2024 Aug;34(8):1130–9. doi: 10.1101/gr.279002.124 (PMC11444171; doi:10.1101/gr.279002.124)
Supplement: Supplement 4 [file Supplemental_figure_S4.pdf]

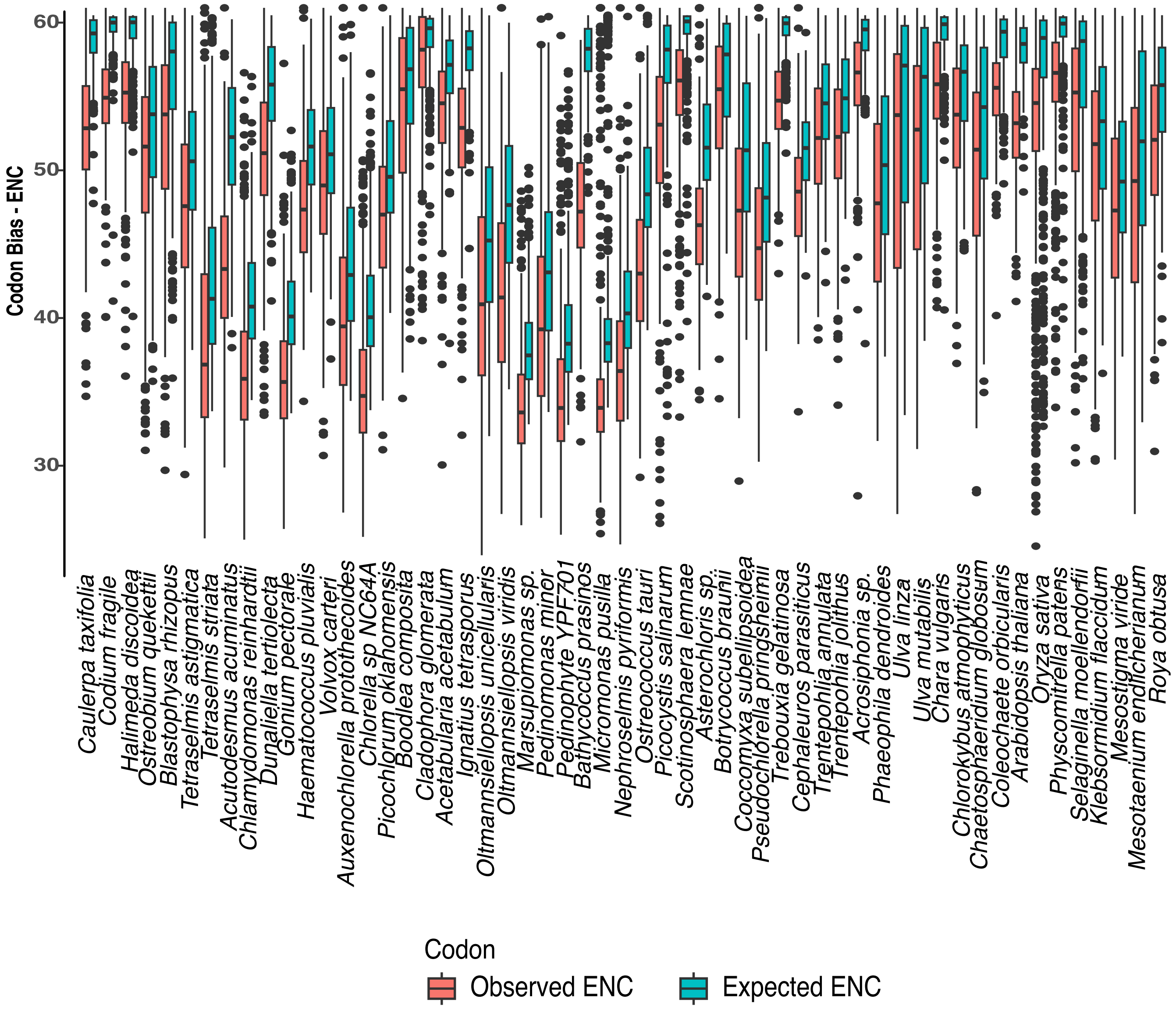


**Supplemental Figure S4:** Paired box plots show the distribution of the observed codon bias (red) and expected codon bias (cyan) for each species across the Green Algal Phylogeny. Codon bias is estimated in terms effective numbers of codons (ENC). This plot shows that observed ENC is lower than the expected ENC across all the species in the green algal phylogeny and suggests the influence translational selection in driving the codon bias.
