# Supplementary material for: Genome-wide patterns of selection–drift variation strongly associate with organismal traits across the green plant lineage
Source: Genome Res. 2024 Aug;34(8):1130–9. doi: 10.1101/gr.279002.124 (PMC11444171; doi:10.1101/gr.279002.124)
Supplement: Supplement 5 [file Supplemental_figure_S5.pdf]

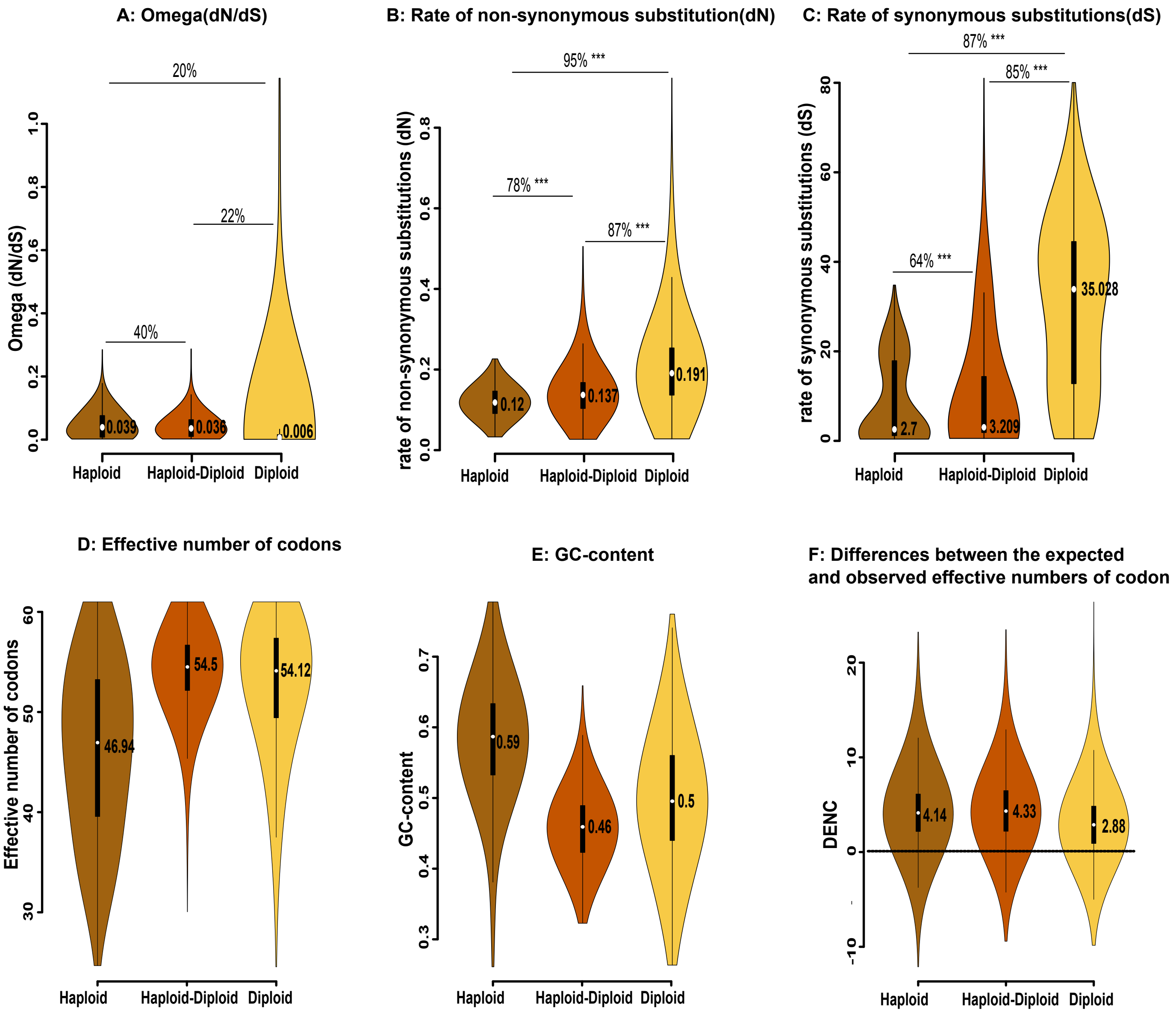

Supplemental Figure S5: Association of life cycle types with molecular evolution, based on the liberal dataset described in the text. The violin plots show the distribution of omega (A), rates of non-synonymous substitutions (B), rates of synonymous substitutions (C), effective number of codons (D), GC content (E) and the difference between the expected and observed ENC (F) for Haploid, Haploid-Diploid and Diploid lineages. The horizontal lines refer to comparisons across body architecture types, with indications of the percentages of genes following expected patterns in these comparisons, and the significance of the differences (*/**/*** for ≤0.05/≤0.001/≤0.0001) as given by p-values of the gene-by-gene Wilcoxon test. The lower values of dN, dS and ENC suggests stronger selection intensity in Haploid relative to Haploid-Diploid and Diploid algal lineages.
