# Supplementary material for: Genome-wide patterns of selection–drift variation strongly associate with organismal traits across the green plant lineage
Source: Genome Res. 2024 Aug;34(8):1130–9. doi: 10.1101/gr.279002.124 (PMC11444171; doi:10.1101/gr.279002.124)
Supplement: Supplement 6 [file Supplemental_figure_S6.pdf]

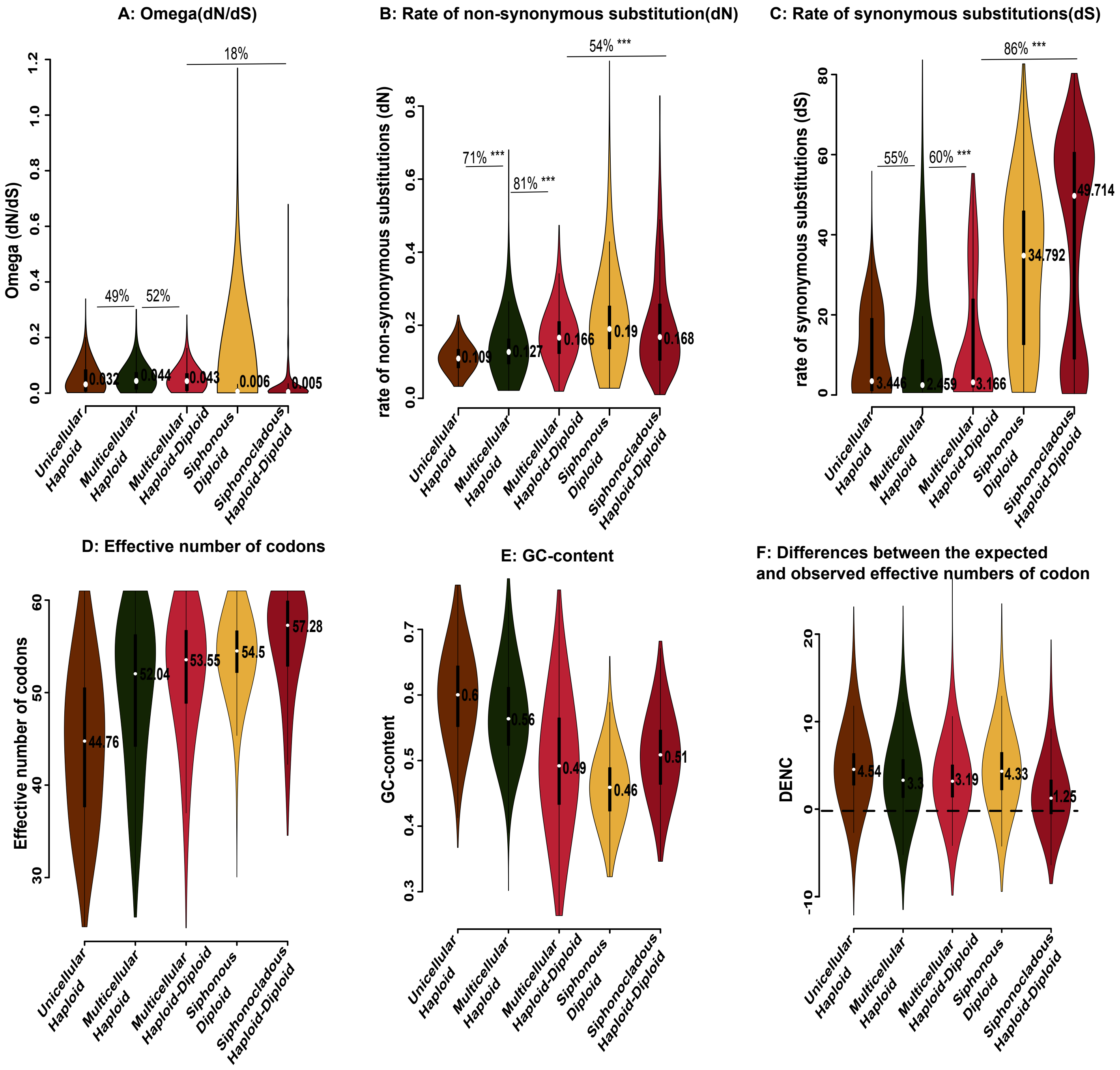

Supplemental Figure S6: Combined effect of body architecture and life cycle types on molecular evolution, based on the liberal dataset described in the text. The violin plots show the distribution of omega (A), rates of non-synonymous substitutions (B), rates of synonymous substitutions (C), effective number of codons (D), GC content (E) and the difference between the expected and observed ENC (F) for five body architecture and life cycle combinations. The horizontal lines refer to comparisons across body architecture types, with indications of the percentages of genes following expected patterns in these comparisons, and the significance of the differences (*/**/*** for ≤0.05/≤0.001/≤0.0001) as given by p-values of the gene-by-gene Wilcoxon test. Significant differences between categories with the same life cyle type and different body architecture suggests that body architecture is the main driver of differences.
