# Supplementary material for: Genome-wide patterns of selection–drift variation strongly associate with organismal traits across the green plant lineage
Source: Genome Res. 2024 Aug;34(8):1130–9. doi: 10.1101/gr.279002.124 (PMC11444171; doi:10.1101/gr.279002.124)
Supplement: Supplement 7 [file Supplemental_figure_S7.pdf]

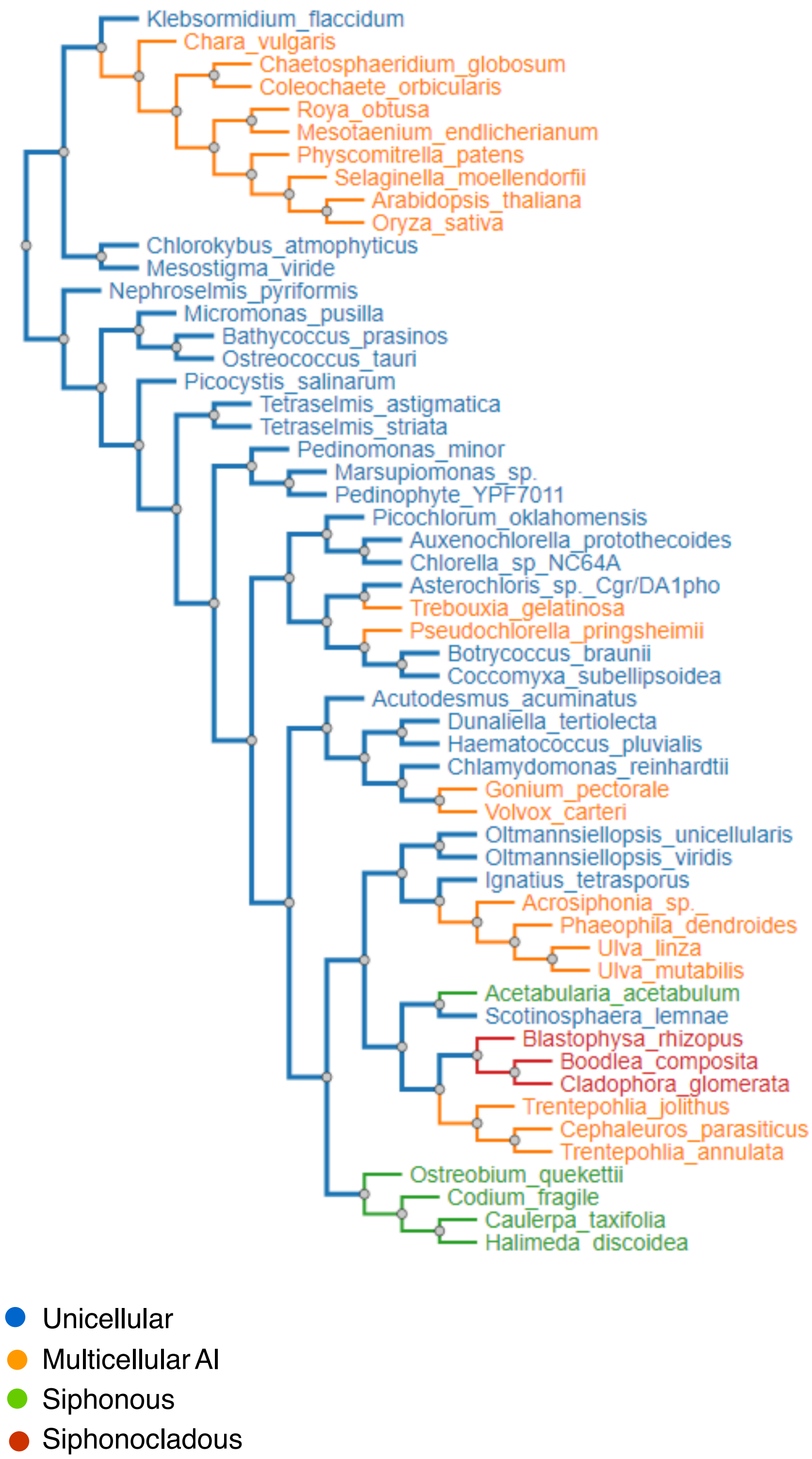

Supplemental Figure S7: Green Algal Phylogeny showing the trait categories (Unicellular, Multicellular, Siphonous and Siphonocladous) for body architecture based model[M1]. The molecular evolutionary traits: omega(dN/dS), non-synonymous(dN) and synonymous(dS) were obtained for each of the four categories by allowing four different selection pattern corresponding to the trait categories.
