# Supplementary material for: Genome-wide patterns of selection–drift variation strongly associate with organismal traits across the green plant lineage
Source: Genome Res. 2024 Aug;34(8):1130–9. doi: 10.1101/gr.279002.124 (PMC11444171; doi:10.1101/gr.279002.124)
Supplement: Supplement 8 [file Supplemental_figure_S8.pdf]

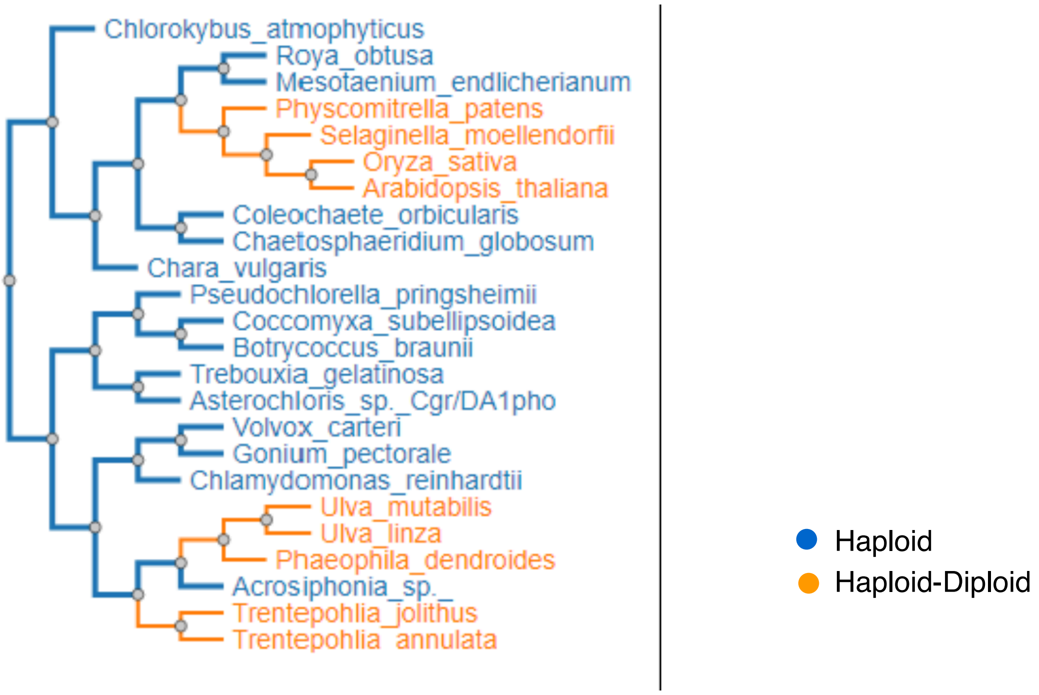

Supplemental Figure S8: Green Algal Phylogeny of conservative dataset showing the trait categories(Haploid and Haploid-Diploid) for Life cycle based model[M2]. The molecular evolutionary traits: omega(dN/dS), non-synonymous(dN) and synonymous(dS) were obtained for each of the two categories by allowing two different selection pattern corresponding to the trait categories.
