# Supplementary material for: Genome-wide patterns of selection–drift variation strongly associate with organismal traits across the green plant lineage
Source: Genome Res. 2024 Aug;34(8):1130–9. doi: 10.1101/gr.279002.124 (PMC11444171; doi:10.1101/gr.279002.124)
Supplement: Supplement 9 [file Supplemental_figure_S9.pdf]

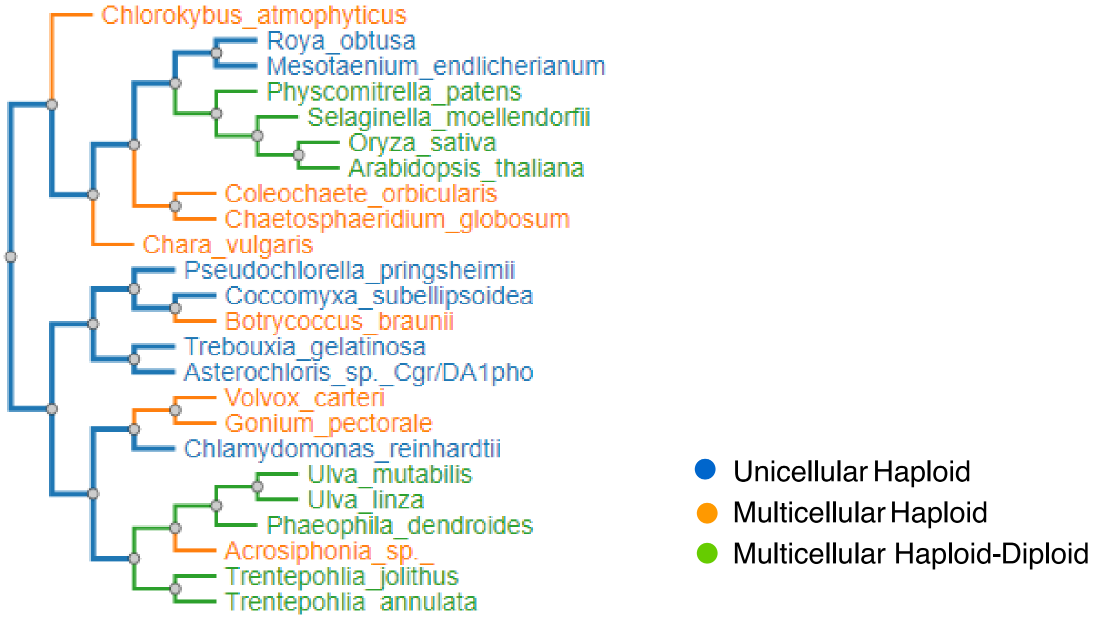

Supplemental Figure S9: Green Algal Phylogeny of conservative dataset showing the trait categories (Unicellular haploid, Multicellular haploid and Multicellular haploid-diploid) for combined model(Body architecture+Life cycle based model[M3]. The molecular evolutionary traits: omega(dN/dS), non-synonymous(dN) and synonymous(dS) were obtained for each of the three categories by allowing three different selection pattern corresponding to the trait categories.
