# Supplementary material for: Genome-wide patterns of selection–drift variation strongly associate with organismal traits across the green plant lineage
Source: Genome Res. 2024 Aug;34(8):1130–9. doi: 10.1101/gr.279002.124 (PMC11444171; doi:10.1101/gr.279002.124)
Supplement: Supplement 10 [file Supplemental_figure_S10.pdf]

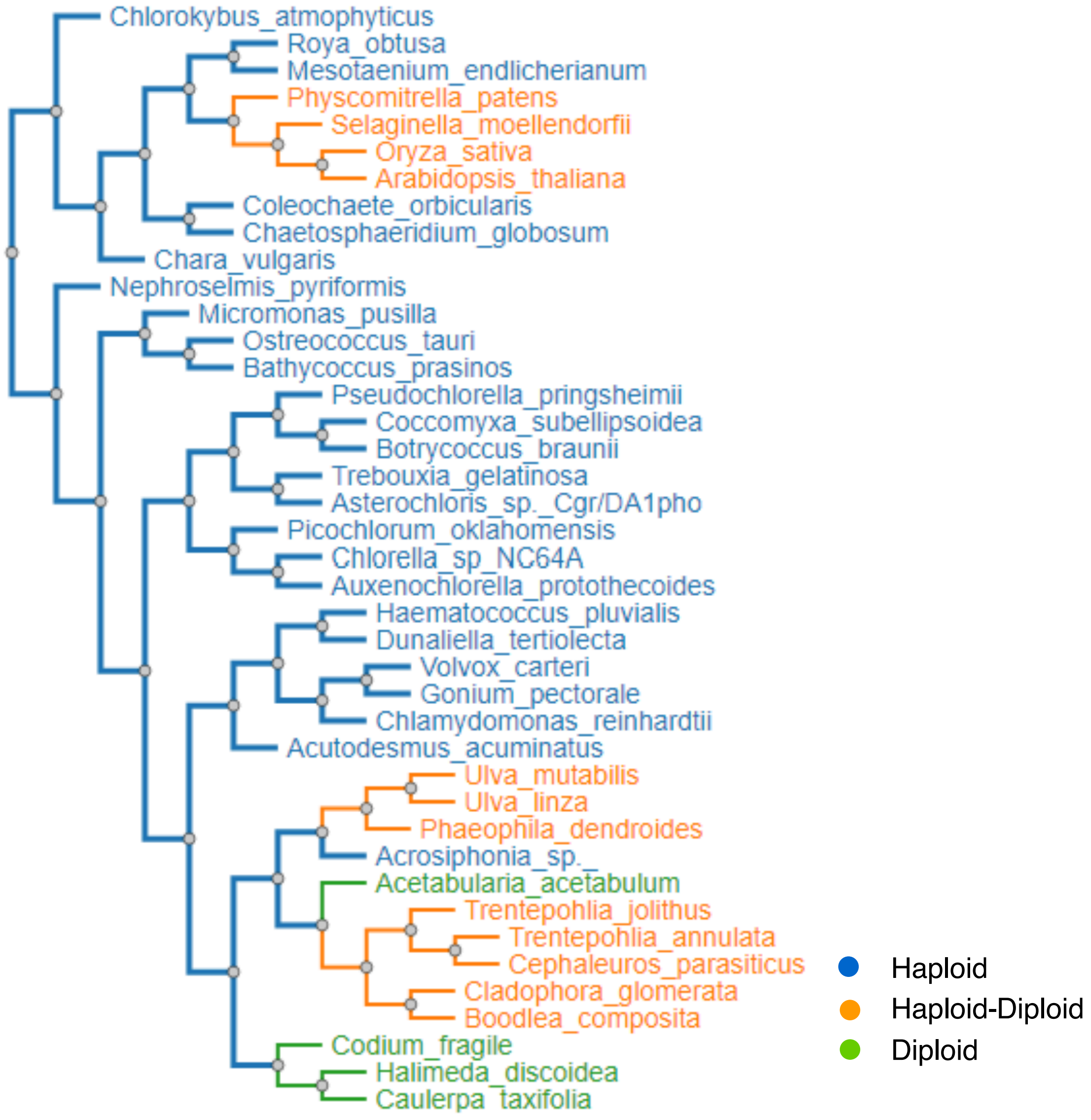

Supplemental Figure S10: Green Algal Phylogeny of liberal dataset showing the trait categories (haploid, haploid-diploid and diploid) for Life cycle based model [M2]. The molecular evolutionary traits: omega (dN/dS), non-synonymous (dN) and synonymous (dS) were obtained for each of the three categories by allowing three different selection pattern corresponding to the trait categories.
