# Supplementary material for: Genome-wide patterns of selection–drift variation strongly associate with organismal traits across the green plant lineage
Source: Genome Res. 2024 Aug;34(8):1130–9. doi: 10.1101/gr.279002.124 (PMC11444171; doi:10.1101/gr.279002.124)
Supplement: Supplement 11 [file Supplemental_figure_S11.pdf]

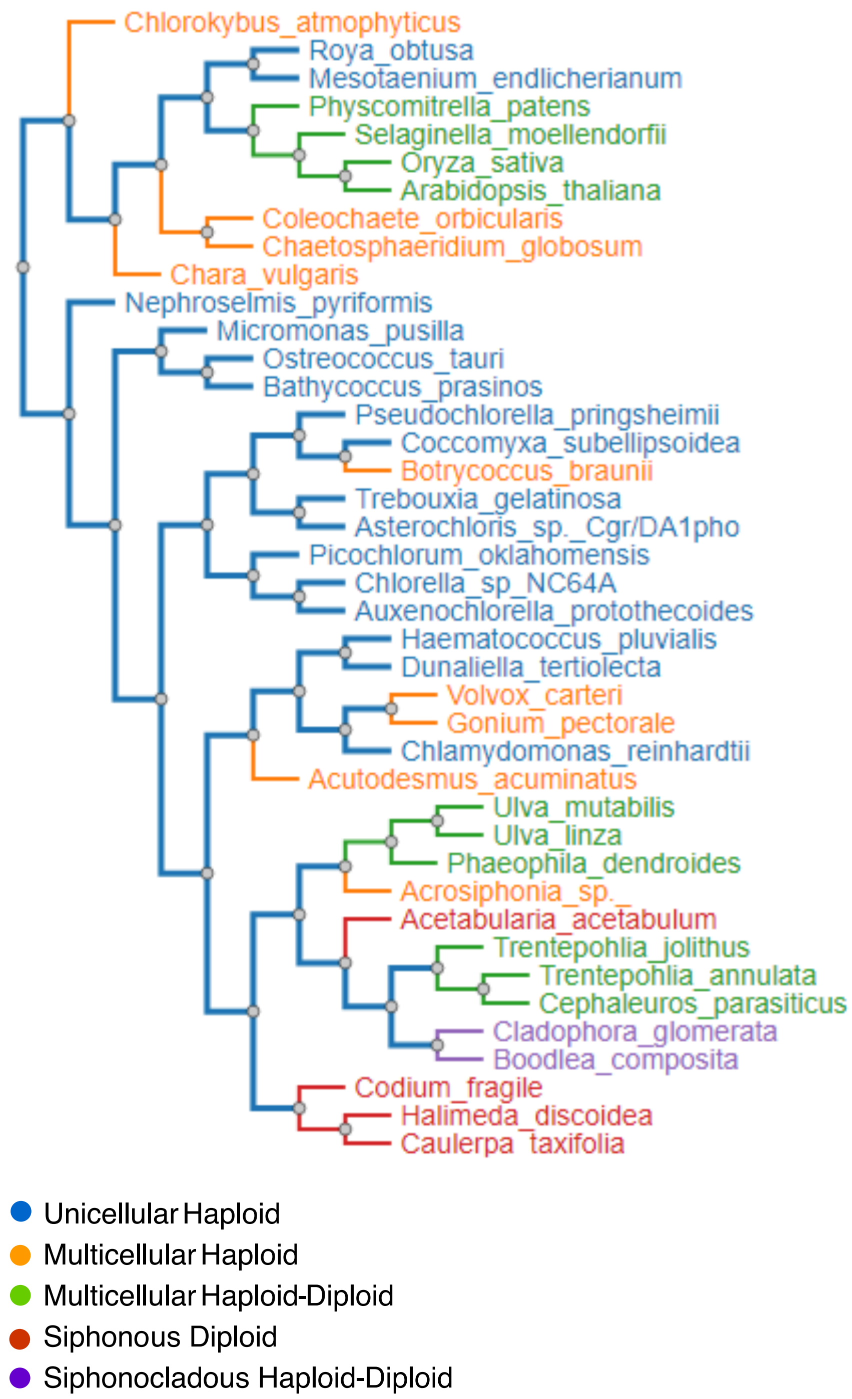

Supplemental Figure S11: Green Algal Phylogeny of liberal dataset showing the trait categories (unicellular haploid, multicellular haploid, multicellular haploid-diploid, siphonocladous haploid-diploid and siphonous diploid) for combined model (Body architecture + Life cycle) [M3]. The molecular evolutionary traits: omega (dN/dS), non-synonymous (dN) and synonymous (dS) were obtained for each of the five categories by allowing five different selection pattern corresponding to the trait categories.
