# Supplementary material for: Genome-wide patterns of selection–drift variation strongly associate with organismal traits across the green plant lineage
Source: Genome Res. 2024 Aug;34(8):1130–9. doi: 10.1101/gr.279002.124 (PMC11444171; doi:10.1101/gr.279002.124)
Supplement: Supplement 12 [file Supplemental_text_S1.docx]

**Supporting Information for**

Genome-wide patterns of selection-drift variation strongly associate with organismal traits across the green plant lineage (Viridiplantae)

**Kavitha Uthanumallian^1*^, Andrea Del Cortona^2^, Susana M. Coelho^3^, Olivier De Clerck^2^, Sebastian Duchene^4^ and Heroen Verbruggen^1*^**

*corresponding authors: Kavitha Uthanumallian, Heroen Verbruggen

**E-mail**: kavitha.uthanumalli@student.unimelb.edu.au, [heroen@unimelb.edu.au](mailto:heroen@unimelb.edu.au)

**This supporting material includes:** Supplemental text S1

**Supplemental text S1**

**Selection patterns for Liberal dataset**

**Methods**

As for the life cycle type-based models described in the main text, trait values for inner tree branches were inferred using stochastic mapping, restricting the occurrence of multicellular to unicellular transitions. PAML branch models were used to estimate selection intensity of each category. Nonparametric Wilcoxon signed ranked test (pairwise) were used to verify differences between categories.

**Life cycle based model**

For life cycle based models the categories are: haploid (abbr H, N=25), diploid (D, N=4) and haploid-diploid (HD, N=12)(Supplemental Figure S7).

*Hypothesis*: Based on the masking hypothesis, diploid lineages are expected to accumulate more mutations, i.e. haploid lineages will have lower rates of substitutions than diploid lineages.

*Results*: Supplemental figure S2 shows the results for this analysis. Haploid lineages had lower rates of non-synonymous and synonymous substitutions relative to diploid and haploid-diploid lineages. Though it supports the hypothesis, we need to cautious about interpreting this as impacts of lifecycles as all the diploid lineages are siphonous algae.

**Combined Life cycle + Architecture based model**

For this model the categories included are as follows: unicellular haploid (UH, N=16), multicellular haploid (MH, N=9), multicellular haploid-diploid (MHD, N=10), siphonous diploid (SpD, N=4) and siphonocladous haploid-diploid (ScHD, N=2) (Supplemental Figure S8). We compared relatable categories like algae with similar life cycle and different architecture [unicellular haploid – multicellular haploid, multicellular haploid-diploid – siphonocladous haploid-diploid] and with similar architecture and different life cycles [multicellular haploid – multicellular haploid-diploid].

*Hypothesis*: We expect the difference in selection patterns between the categories with similar life cycle and different architecture [UH < MH, MHD < ScHD] can mainly to be due the impacts of body architecture as they have same life cycle. Accordingly, multicellular haploid - multicellular haploid-diploid algae can possibly highlight the impacts of lifecycles as they belong to the same body architectures category.

*Results*: Supplemental figure S3 shows the results for this analysis. Life cycle and architecture based model on liberal dataset shows that between unicellular and multicellular with haploid life cycles (UH-MH), lower *d*_N_, ENC and higher GC content suggests stronger purifying selection in unicellular haploid (Supplemental Figure S3:B,D,E), supporting that body architecture impacts molecular evolution. Similarly, multicellular haploid-diploid with lower *d*_N_, *d*_S_, ENC and higher GC than siphonocladous haploid-diploid, further support the impacts of body architecture (Supplemental Figure S3:B,C,D,E).

Though lower *d*_N_, *d*_S_, ENC and higher GC in multicellular haploid than multicellular haploid-diploid support life cycle impacts we need to cautious here as many multicellular haploid are small algae like Gonium, Volvox, Botrycoccus and other hand multicellular haploid-diploid are Trentepohliales, ulvophytes and streptophytes.

**Conclusions**

Results based on liberal datasets supports the conclusions derived from the conservative dataset. We can infer that body architecture impacts the molecular evolution of algal lineages, while still the impact of the life cycle types on the molecular evolution of green algae appears limited.
